# Chordoid Meningioma: The Rarest Subtype of Grade II Meningioma

**DOI:** 10.7759/cureus.74084

**Published:** 2024-11-20

**Authors:** Jose Valerio, Noe Santiago Rea, Jorge Zumaeta, Luis Rey Martinez

**Affiliations:** 1 Neurological Surgery, Palmetto General Hospital, Hialeah, USA; 2 Neuro-Oncology, Miami Neuroscience Center at Larkin, Miami, USA; 3 Neurosurgical Oncology, Latinoamerica Valerio Foundation, Weston, USA; 4 Neurosurgery Service, Hospital Nacional Arzobispo Loayza, Lima, PER; 5 Servicio de Neurocirugía Vascular, Tumores y Funcional, Neurocirugía, Hospital Nacional Guillermo Almenara Irigoyen, Lima, PER; 6 Pathology and Laboratory Medicine, Palmetto General Hospital, Hialeah, USA

**Keywords:** brain edema, diagnosis, intracranial meningioma, neurosurgery, pathology

## Abstract

Chordoid meningioma, a rare WHO grade II tumor, is known for its aggressive behavior and high recurrence rate. We report a case of a 44-year-old woman with progressive left-sided weakness, where imaging revealed a 3.0 cm lesion in the right sphenoidal wing with significant midline shift and edema. Following corticosteroid treatment, the patient underwent embolization and complete tumor resection, achieving full motor recovery. Pathology confirmed a chordoid meningioma. Gross total resection is vital to minimize the risk of recurrence, while subtotal resection is linked to higher recurrence, often with elevated MIB-1 scores. Adjuvant radiotherapy may be used in high-risk cases, though it carries a risk of malignant transformation. Early detection, complete resection, and close follow-up are key to optimal outcomes.

## Introduction

The most common benign intracranial tumor is meningioma [1-3], accounting for 13 to 36% of all primary central nervous system neoplasms. Around 90% of all meningiomas are benign and classified as WHO grade I. After 2007, the incidence of meningioma grade II increased from 5% to 20-35% since all meningiomas invading the cortex are now considered grade II [2,3]. Grade II meningiomas are classified as malignant lesions [2,4] because they are more aggressive and more prone to recurrence [1,4,5]; chordoid meningioma is the rarest subtype [3].

Kepes et al. defined chordoid meningioma due to its chordoma-like pathologic features [1]. Its incidence has been reported to be 0.32 to 1.0% of all intracranial meningiomas [1,4]. Given this low incidence, the clinical, radiological, pathological, and prognostic features of chordoid meningioma remain unclear and controversial [1].

Numerous studies have investigated imaging features to differentiate low-grade from high-grade meningiomas. Factors like larger tumor size and irregular shape are associated with higher-grade meningiomas but lack specificity, particularly for atypical or anaplastic types. Advanced MRI techniques, including diffusion, perfusion, and MR spectroscopy, have been explored to address this. Among these, the apparent diffusion coefficient (ADC) is the most promising for distinguishing WHO grades. Strong correlations between ADC metrics and tumor histology have been shown, but only one study with four patients has specifically evaluated ADC for identifying chordoid meningiomas [5].

Accordingly, we conducted a comprehensive literature review on this topic concerning a surgically treated case that demonstrated a favorable postoperative progression.

## Case presentation

**Figure 1:**
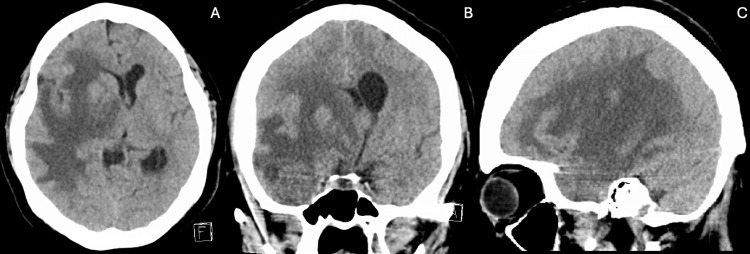
Right sphenoid wing meningioma observed on non-contrast brain CT scan. Non-contrast brain CT in axial (A), coronal (B), and sagittal (C) views demonstrates a lesion located at the right temporal pole with significant perilesional vasogenic edema, causing a severe midline shift of over 1 cm.

**Figure 2:**
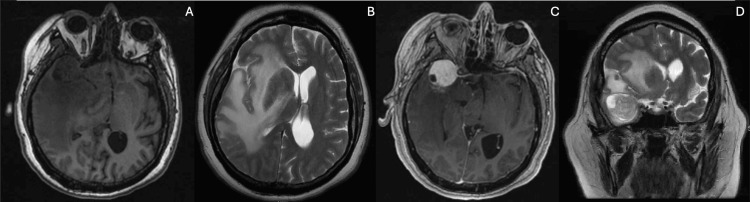
Right sphenoid wing meningioma observed on contrasted MRI. There is a rounded lesion at the level of the right sphenoid wing. It appears isointense and hypointense on T1 (A), and noticeable vasogenic perilesional edema is visible on T2 (B), causing a midline shift of more than 1 cm. The lesion shows intense and homogeneous enhancement with contrast (C). The midline shift caused by the vasogenic edema due to the right sphenoid wing meningioma is more clearly observed (D).

**Figure 3:**
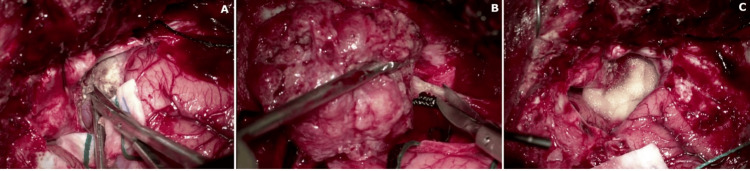
Intraoperative images of intracranial meningioma resection. Intraoperative image illustrating the devascularization and detachment of the tumor from its implantation site (A), the en bloc removal of the tumor (B), and the final appearance of the surgical bed after resection (C).

**Figure 4:**
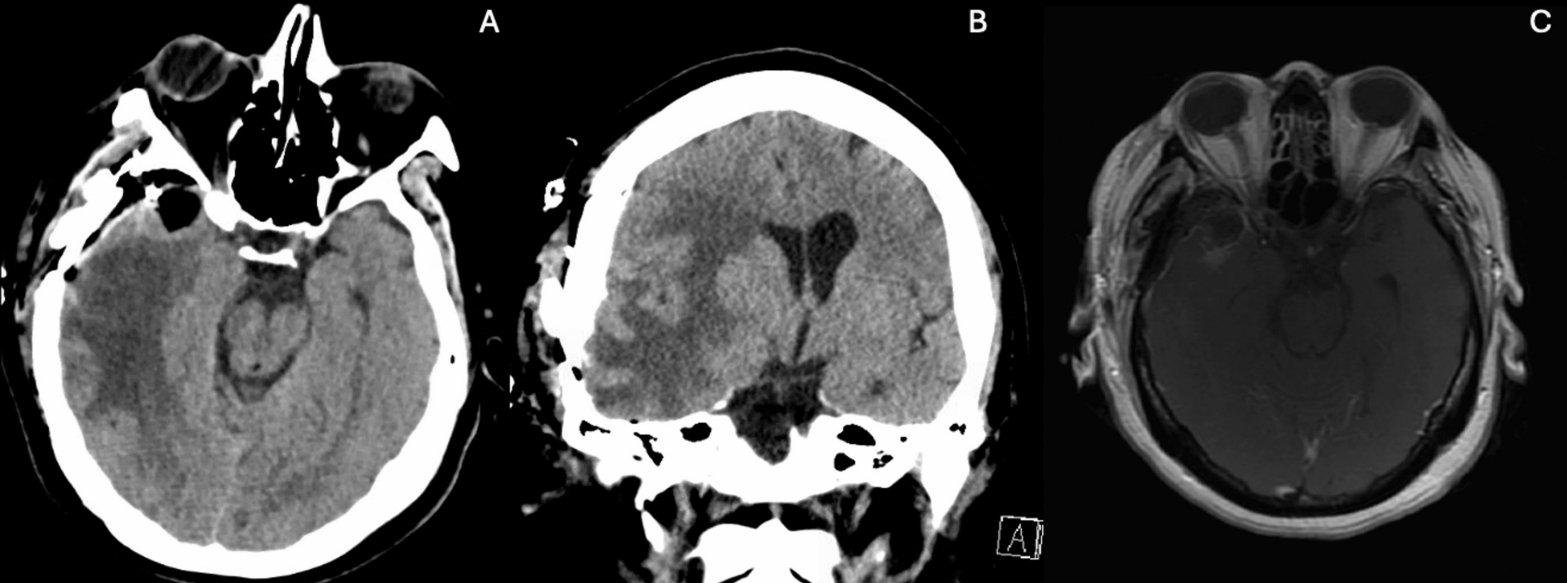
Postoperative images after intracranial meningioma resection. Immediate postoperative CT shows the surgical bed with the meningioma resected without complications (A), along with partial recovery of the midline and expansion of the previously collapsed right lateral ventricle (B). Contrast-enhanced MRI demonstrates gross total resection of the meningioma (C).

**Figure 5:**
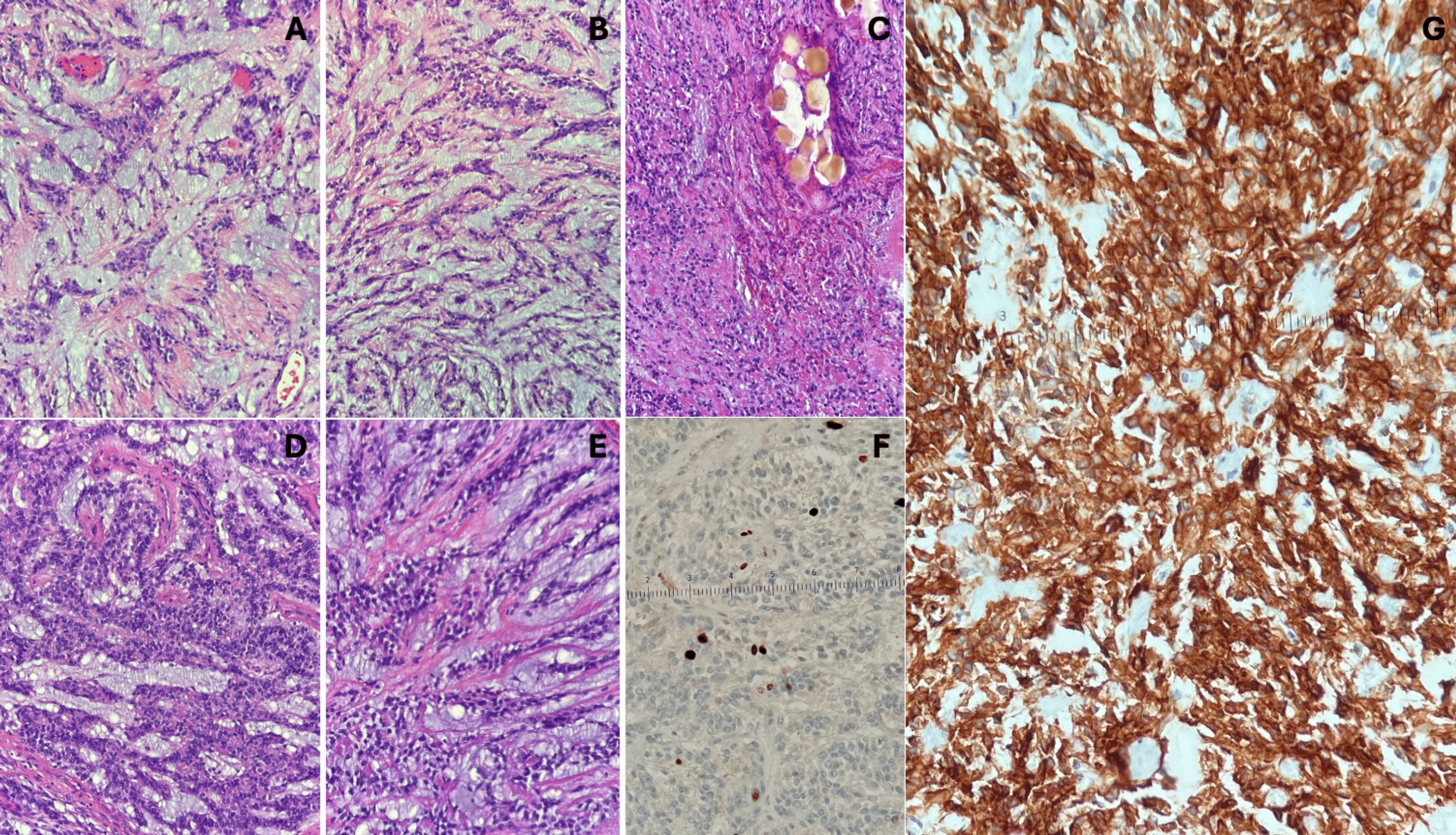
Confirmatory histological sections of chordoid meningioma. Cords of small to intermediate-sized epithelioid cells embedded within a mucin-rich matrix (A and B). Tumor cells adjacent to a vessel with embolization microspheres are shown in (C). Cords and trabeculae of small to intermediate and variably vacuolated cells in a mucin-rich matrix appear in (D and E). Tumor cells with a low (~2%) Ki-67 index are presented in (F). Somatostatin receptor subtype 2A (SSTR2A) immunoreactive tumor cells are depicted in (G).

A 44-year-old female with a medical history of hypertension, hyperlipidemia, type 2 diabetes mellitus, and schizophrenia came to the hospital with complaints of progressive weakness. An initial CT scan of the brain (Figure 1) showed a 1 cm midline shift from right to left and signs of subfalcine herniation. A subsequent magnetic resonance imaging (Figure 2) revealed a 3.0x3.0x3.0 cm enhancing lesion in the middle third of the right sphenoid wing, raising concerns about a possible tumor, such as a meningioma. The lesion was associated with significant perilesional vasogenic edema, resulting in a progression of the midline shift to 1.4 cm. The physical exam showed the patient was awake, alert, and oriented to time, space, and person. Cranial nerves I to XII showed no alteration. Strength in the upper and lower left extremities was 4/5, while in the upper and lower right extremities, it was 5/5. Sensation was intact for superficial and deep pain, tactile sensation, and discrimination. Reflexes were intact in both upper and lower extremities at 2/4. The patient’s management began with intravenous corticosteroids, followed by right middle meningeal artery embolization, right temporal craniotomy, and resection (Figure 3) of the meningioma. After tumor resection, the patient complained only of headaches; nausea and vomiting were not present. Motor strength recovered to 5/5 bilaterally in the upper and lower extremities. Control imaging studies were requested (Figure 4). Pathology results confirmed a chordoid meningioma (Figure 5), which was SSTR-2 positive, while D2-40, Pankeratin, CK20, EMA&PR, and S-100 were negative. Ki-67 was positive in 3-4% of cells. 

## Discussion

Meningiomas are the most common type of primary intracranial tumors. While most of these tumors are benign, those classified as grades II and III can invade brain tissue and have a higher rate of recurrence [3]. Among the grade II histological types, chordoid meningioma is the rarest, which makes understanding its clinical characteristics and management [2] especially important due to their higher recurrence rates [3]. Chordoid meningiomas account for 0.5% of all meningiomas; until 2022, fewer than 300 cases had been reported worldwide [2].

The most common symptom of chordoid meningiomas is headaches, although no specific objective signs have been identified in the literature [6]. The presence of motor deficits or seizures depends on the tumor’s location and the pressure it exerts on a particular brain region [3].

According to Jie D et al., the most common locations for chordoid meningiomas are the skull base, followed by convexity and the ventricle. However, other studies have found that convexity chordoid meningiomas are more common than those at the skull base [1]. The location of a meningioma significantly influences the extent of resection. Tumors located outside the middle convexity are generally easier to completely remove [7]. Peritumoral edema has been described as a prognostic factor in meningiomas [4].

In this case report, the primary symptom was not headache; the most prominent clinical feature was significant left-sided weakness. The lesion was located at the skull base, specifically in the mid-third of the right sphenoid wing, and exhibited a notable mass effect primarily due to severe perilesional vasogenic edema, rather than the size of the lesion itself.

The most important prognostic factor in chordoid meningioma is the extent of resection. Therefore, the goal should be gross total resection. However, the recurrence rate remains high when only subtotal resection is performed. This was evidenced by one study in which all patients with a subtotal resection experienced tumor recurrence, while only 1 out of 29 patients had a recurrence after gross total resection [2,6]. In our case, we successfully achieved complete gross total resection, which was confirmed by a follow-up MRI.

As previously mentioned, chordoid meningiomas have a high risk of recurrence, primarily related to Simpson grades I-II. Likewise, recent reports suggest that tumors with a high MIB-L1 score possess a higher risk of recurrence [2,4]. Our case exhibited a Ki-67 index of 3-4%, which is considered a threshold value for low scores. Even though adjuvant radiotherapy is recommended for patients with subtotal resection and high MIB-L1 scores, some studies assert that radiotherapy plays a role in malignant transformation [4].

Chordoid meningiomas are identified by their histopathological characteristics, which include spindled-to-epithelioid cells with eosinophilic cytoplasm. These cells are organized in chains and cords within a basophilic, myxoid extracellular matrix. The stromal component is notably rich in acidic mucopolysaccharides and shows positive staining with mucicarmine, periodic acid-Schiff, and Alcian blue [5]. Correctly identifying vacuolated trabeculae of neoplastic cells in a myxoid background accompanied by areas of typical meningioma allows a straightforward diagnosis of chordoid meningioma [2]. In terms of immunohistochemistry, tumoral cells express epithelial membrane antigen (EMA) and vimentin but do not express cytokeratin. This helps to differentiate them from chordomas, which are the main differential diagnosis. Another important differential diagnosis is chordoid gliomas, which intensely express the glial fibrillary acidic protein (GFAP) [6].

## Conclusions

Chordoid meningiomas are rare, accounting for only a small percentage of all meningiomas. They are classified as WHO grade II due to their aggressive nature and high recurrence rate. This case report emphasizes the importance of early detection and aggressive treatment strategies, such as gross total resection, to minimize the risk of recurrence. However, the inherent nature of a case report limits the generalizability of the findings. Although the WHO grade II tumor subtype poses unique challenges, complete resection remains the most crucial prognostic factor. The patient in this case demonstrated a favorable outcome post-surgery, with a resolution of symptoms and no immediate signs of recurrence. Given the high recurrence rate, especially in cases with subtotal resection or a high MIB-L1 score, close follow-up and consideration of adjuvant therapies are essential.
